# Changes in total and differential leukocyte counts during the clinically silent liver phase in a controlled human malaria infection in malaria-naïve Dutch volunteers

**DOI:** 10.1186/s12936-017-2108-1

**Published:** 2017-11-10

**Authors:** Marlies E. van Wolfswinkel, Marijke C. C. Langenberg, Linda J. Wammes, Robert W. Sauerwein, Rob Koelewijn, Cornelus C. Hermsen, Jaap J. van Hellemond, Perry J. van Genderen

**Affiliations:** 1 Institute for Tropical Diseases, Harbour Hospital, Rotterdam, The Netherlands; 2 Department of Medical Microbiology and Infectious Diseases, Erasmus MC and Harbour Hospital, Rotterdam, The Netherlands; 3 Department of Parasitology, Leiden University Medical Center, Leiden, The Netherlands; 4 Department of Medical Microbiology, Radboud University Medical Center, Nijmegen, The Netherlands

**Keywords:** Controlled Human Malaria Infection, *Plasmodium falciparum*, Leukocyte count, Lymphocyte count, Lymphocytopenia, Neutropenia, Monocytes, Neutrophil to lymphocyte count ratio, Liver phase

## Abstract

**Background:**

Both in endemic countries and in imported malaria, changes in total and differential leukocyte count during *Plasmodium falciparum* infection have been described. To study the exact dynamics of differential leukocyte counts and their ratios, they were monitored in a group of healthy non-immune volunteers in two separate Controlled Human Malaria Infection (CHMI) studies.

**Methods:**

In two CHMI trials, CHMI-a and CHMI-b, 15 and 24 healthy malaria-naïve volunteers, respectively, were exposed to bites of infected mosquitoes, using the *P. falciparum* research strain NF54 and the novel clones NF135.C10 and NF166.C8. After mosquito bite exposure, twice-daily blood draws were taken to detect parasitaemia and to monitor the total and differential leukocyte counts. All subjects received a course of atovaquone–proguanil when meeting the treatment criteria.

**Results:**

A total of 39 volunteers participated in the two trials. Thirty-five participants, all 15 participants in CHMI-a and 20 of the 24 volunteers in CHMI-b, developed parasitaemia. During liver stage development of the parasite, the median total leukocyte count increased from 5.5 to 6.1 × 10^9^ leukocytes/L (p = 0.005), the median lymphocyte count from 1.9 to 2.2 (p = 0.001) and the monocyte count from 0.50 to 0.54 (p = 0.038). During the subsequent blood stage infection, significant changes in total and differential leukocyte counts lead to a leukocytopenia (nadir median 3.3 × 10^9^ leukocytes/L, p = 0.0001), lymphocytopenia (nadir median 0.7 × 10^9^ lymphocytes/L, p = 0.0001) and a borderline neutropenia (nadir median 1.5 × 10^9^ neutrophils/L, p = 0.0001). The neutrophil to lymphocyte count ratio (NLCR) reached a maximum of 4.0. Significant correlations were found between parasite load and absolute lymphocyte count (p < 0.001, correlation coefficient − 0.46) and between parasite load and NLCR (p < 0.001, correlation coefficient 0.50). All parameters normalized after parasite clearance.

**Conclusions:**

During the clinically silent liver phase of malaria, an increase of peripheral total leukocyte count and differential lymphocytes and monocytes occurs. This finding has not been described previously. This increase is followed by the appearance of parasites in the peripheral blood after 2–3 days, accompanied by a marked decrease in total leukocyte count, lymphocyte count and the neutrophil count and a rise of the NLCR.

**Electronic supplementary material:**

The online version of this article (10.1186/s12936-017-2108-1) contains supplementary material, which is available to authorized users.

## Background

Controlled Human Malaria Infection (CHMI) is a well-established clinical model that was developed for the evaluation of candidate malaria vaccines and drugs [1]. In this model, healthy volunteers are infected by malaria sporozoites via exposure to the bites of infected mosquitoes or via inoculation by needle and syringe [2]. CMHI studies have traditionally been performed using the *Plasmodium falciparum* strain NF54, which is thought to originate from Africa [3]. More recently, the clones NF135.C10, and NF166.C8, originating from *P. falciparum* strains from Cambodia and Guinea, respectively, have been added to the CHMI portfolio [4–6]. In the present study, the CHMI model is used to study total and differential leukocyte count changes and their ratios during the liver phase and blood phase of malaria.

Changes in total and differential leukocyte count during *P. falciparum* infection have been described in both clinical studies and CHMI studies previously. In clinical studies, both in endemic countries and in patients with imported malaria [7–10], the most pronounced change is the decrease of peripheral lymphocytes. Lymphocytopenia has been observed in 45 to 63% of patients with an imported *P. falciparum* infection, but was less prominent in patients with some degree of anti-malarial immunity [11, 12]. In a recent large cross-sectional study on leukocyte count changes in returning travellers, malaria was the second most common cause of absolute lymphocytopenia [13]. Differential leukocyte count changes have also been studied in patients with imported malaria. The neutrophil-to-lymphocyte count ratio (NLCR), which was introduced by Zahorec et al. as a parameter of systemic inflammation in critically ill surgical and medical patients [14], correlated with parasitaemia and normalized after parasite clearance in patients with imported malaria [11, 12]. One study showed correlations between parasitaemia and both the monocyte to lymphocyte count ratio (MLCR) and neutrophil to monocyte count ratio (NMCR) [11].

In CHMI studies, decreases in total leukocyte count, neutrophil count and lymphocyte count have been described during the blood phase [15–20], but could not evaluate leukocyte changes during liver stages of the parasite life cycle.

In the present study, the changes of differential leukocyte counts and their ratios were monitored during the liver phase and the development of detectable blood parasitaemia in a group of healthy non-immune volunteers in the CHMI model using the NF54, NF166.C8 and NF135.C10 *P. falciparum* clones.

## Methods

### Study design

The present study was performed using two Controlled Human Malaria Infection studies, CHMI-a and CHMI-b [4]. Healthy malaria-naïve adult Dutch volunteers were recruited at the Harbour Hospital, Rotterdam, after signing informed consent. In CHMI-a, fifteen subjects were randomly allocated to three groups of n = 5, to be infected by bites of five mosquitoes per subject carrying either the NF54 strain of *P. falciparum*, the NF135.C10 clone or the novel NF166.C8 clone. In CHMI-b, 24 subjects were randomly allocated to six groups of n = 4, to bites by one, two or five mosquitoes carrying either NF135.C10 or NF166.C8. From day 5 after exposure, subjects were seen in clinic twice daily for registration of vital parameters and adverse events and for venous blood draws for thick blood smear, qPCR, and a wide range of laboratory parameters including differential leukocyte counts. *Plasmodium falciparum* parasitaemia was quantified by qPCR as described before [21]. In CHMI-a, subjects were treated with atovaquone–proguanil 1000/400 mg daily for 3 days upon their first positive thick blood smear, defined as ≥ 2 parasites per 225 high-powered fields (equivalent to 0.5 µL blood). In CHMI-b, subjects were treated with the same regimen as soon as 2 consecutive blood samples were positive by qPCR, defined as > 500 parasites/mL. This change in treatment initiation between the two studies was implemented to minimize safety risks following a cardiac serious adverse event in a previous study [15, 22]. Daily thick blood smears and qPCR were continued after treatment until complete clearance of asexual parasites.

### Data selection

This study focused on leukocyte count changes during the liver stage and blood stage of the malaria infection in the participants of CHMI-a and CHMI-b. Data from the measurements taken during the liver phase in both studies were combined. However, the described differences in methodology regarding the initiation of treatment in both studies did not permit combination of blood phase CHMI-a and CHMI-b data. For this phase, the data of only CHMI-b were used, as in this study the subjects who remained qPCR negative, but received the same treatment as the parasitaemic subjects, could be used as a negative control group to exclude an effect of the atovaquone–proguanil on the evaluated parameters.

### Statistical analysis

To determine whether the parameters of interest changed over time a Friedman test was performed. Next, a more detailed analysis of the timing of these changes was done using a linear model. Correlations were tested with the Spearman’s rank-order correlation. Data were not normally distributed and given as median (interquartile range) unless stated otherwise.

### Definitions

Leukocytopenia: Leukocyte count < 4.0 × 10^9^/L;
Lymphocytopenia: Lymphocyte count < 1.0 × 10^9^/L;
Neutropenia: Neutrophil count < 1.5 × 10^9^/L.
Day of treatment (DT): The day the subject started a 3-day course of atovaquone–proguanil, directly upon meeting the treatment criteria. The days from 2 days before until 3 days after this day are named DT − 2 to DT + 3.
Liver phase: The subjects who eventually developed parasitaemia were regarded as being in the liver phase on day 5 and 6 of the study protocol, when the peripheral blood samples of all subjects were still qPCR negative.
Detectable blood parasitaemia: Detectable blood parasitaemia is defined as qPCR positive, a method with a detection limit of 50 parasites per mL.

## Results

### Development of parasitaemia and phases of the infection

In CHMI-a, all participants developed parasitaemia, with a median prepatent period of 8 days (range 6.5–10.5 days), determined by thick blood smear, upon which curative treatment was initiated. As determined retrospectively, all subjects were still qPCR negative on day 5 and 6 of the study protocol. Of the 24 participants in CHMI-b, twenty (83%) developed parasitaemia. The median prepatent period as determined by qPCR was 7 days (range 7–9 days) and the median time between exposure and the day the treatment criteria were met (hereafter called day of treatment [DT]) was 8 days (range 7–11 days).

**Fig. 1:**
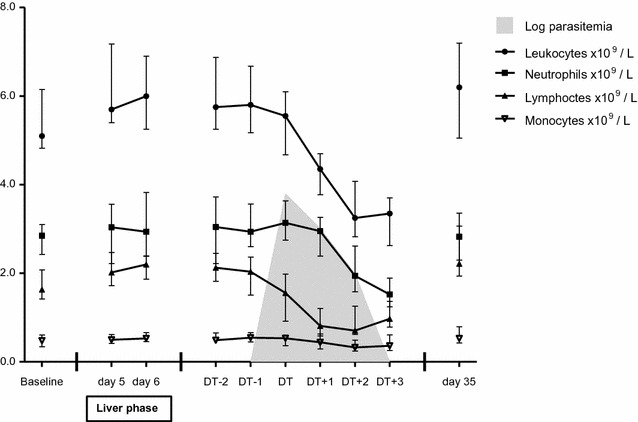
Changes in total and differential leukocyte counts in the 20 subjects who developed malaria in CHMI-b. The data are shown as medians (dots) and interquartile ranges (whiskers). The data from DT − 2 until DT + 3 were synchronized on DT

For CHMI-b, the data from 2 days before DT (DT − 2) until 3 days after DT (DT + 3) were synchronized on DT (Fig. 1). On DT − 2, four subjects had a positive qPCR, all with a low parasitaemia (385–713 parasites/mL) and did not meet the treatment criteria at that moment. The number of parasitaemic subjects increased to nine subjects on DT − 1. The median parasite load on DT, when all patients met the treatment criteria, was 6265 parasites per mL. Hereafter the parasite load declined to 1099 on DT + 1 and 100 parasites per ml on DT + 2.

### Changes in differential leukocyte counts and count ratios during the liver phase

All 35 subjects who developed detectable parasitaemia in CHMI-a and CHMI-b were still qPCR negative at day 5 and 6 of the study protocol, and the infection was therefore regarded as being in the liver phase [23]. During this phase an increase of the total leukocyte count, lymphocyte count and the monocyte count was observed in the 35 subjects who developed parasitaemia later on in CHMI-a and CHMI-b. Changes in neutrophil count and the differential cell count ratios were not significant. No significant changes were observed in the four subjects who remained qPCR negative in CHMI-b. No differences between the total or differential leukocyte counts were found between subjects infected with NF54 strain, the NF135.C10 clone or the NF166.C8 clone (Table 1).

**Table 1:** The course of differential leukocyte counts and their ratios during the liver phase in the subjects who develop malaria

	Leukocytes × 10^9^/L	Neutrophils × 10^9^/L	Lymphocytes × 10^9^/L	Monocytes × 10^9^/L	NLCR	MLCR	NMCR
CHMI-a (n = 15)							
Baseline	5.8 (5.3–6.5)	3.1 (2.9–3.3)	1.9 (1.9–2.3)	0.51 (0.48–0.58)	1.6 (1.4–1.7)	Baseline	5.8 (5.3–6.5)
Day 5	6.8 (6.1–7.3)	3.6 (2.9–4.0)	2.3 (2.0–2.5)	0.55 (0.46–0.63)	1.6 (1.4–1.8)	Day 5	6.8 (6.1–7.3)
Day 6	6.9 (5.8–7.6)	3.2 (2.7–4.1)	2.4 (1.9–2.7)	0.55 (0.49–0.58)	1.3 (1.1–1.9)	Day 6	6.9 (5.8–7.6)
p value	0.105	0.721	0.140	0.208	0.623	p value	0.105
CHMI-b (n = 20)							
Baseline	5.1 (4.9–6.1)	2.8 (2.5–3.1)	1.6 (1.5–2.0)	0.48 (0.35–0.60)	1.8 (1.1–2.0)	0.3 (0.2–0.3)	5.8 (4.7–8.3)
Day 5	5.7 (5.4–7.2)	3.1 (2.2–3.6)	2.0 (1.7–2.5)	0.49 (0.43–0.60)	1.3 (1.0–2.1)	0.2 (0.2–0.3)	5.9 (4.8–7.2)
Day 6	6.0 (5.3–6.9)	3.0 (2.4–3.8)	2.2 (1.9–2.4)	0.53 (0.46–0.65)	1.4 (1.1–1.8)	0.2 (0.2–0.3)	5.3 (4.6–7.1)
p value	0.051	0.195	0.004	0.012	0.165	0.196	0.848
Combined (n = 35)							
Baseline	5.5 (5.0–6.2)	2.9 (2.6–3.2)	1.9 (1.6–2.2)	0.50 (0.38–0.60)	1.7 (1.3–2.0)	0.3 (0.2–0.3)	6.1 (4.8–8.4)
Day 5	6.1 (5.5–7.2)	3.3 (2.5–3.7)	2.2 (1.8–2.5)	0.50 (0.43–0.54)	1.5 (1.1–1.9)	0.2 (0.2–0.3)	6.1 (4.9–8.1)
Day 6	6.1 (5.5–7.0)	3.0 (2.6–3.9)	2.2 (1.9–2.6)	0.54 (0.47–0.62)	1.4 (1.1–1.8)	0.2 (0.2–0.3)	5.7 (4.8–7.2)
p value	0.005	0.413	0.001	0.038	0.121	0.368	0.971

The data of CHMI-a and CHMI-b are shown separately and combined

Data in this table are not normally distributed and shown as median (interquartile range). p values in this table were derived from Friedman tests

### Changes in differential leukocyte counts and count ratios after the liver phase

In the group of twenty parasitaemic subjects in CHMI-b, highly significant changes in the total leukocyte count and differential cell counts of neutrophils, lymphocytes and monocytes were observed in the days before DT and during treatment (Table 2 and Fig. 1). These changes were not seen in non-parasitaemic individuals (Additional file 1: Table S1 and Additional file 2: Figure S1). Of interest, both parasitaemic and non-parasitaemic individuals received anti-malarial treatment, thus excluding an effect of atovaquone–proguanil treatment on the observed changes.

**Table 2:** The course of differential leukocyte counts and their ratios in the 20 subjects who develop malaria in CHMI-b

	Leukocytes × 10^9^/L	Neutrophils × 10^9^/L	Lymphocytes × 10^9^/L	Monocytes × 10^9^/L	NLCR	MLCR	NMCR	Parasite load parasites/mL
Baseline	5.1 (4.9–6.1)	2.8 (2.5–3.1)	1.6 (1.5–2.0)	0.48 (0.35–0.60)	1.8 (1.1–2.0)	0.3 (0.2–0.3)	5.8 (4.7–8.3)	N/A
DT − 2	5.8 (5.4–6.8)	3.0 (2.3–3.7)	2.1 (1.9–2.4)	0.49 (0.43–0.61)	1.4 (1.0–1.7)	0.2 (0.2–0.3)	5.6 (4.3–6.8)	0 (0–0)
DT − 1	5.8 (5.3–6.6)	2.9 (2.6–3.5)	2.0 (1.5–2.3)	0.55 (0.46–0.65)	1.4 (1.1–2.1)	0.3 (0.2–0.3)	5.2 (4.5–6.7)	0 (0–374)
DT	5.6 (4.8–6.1)	3.1 (2.8–3.6)	1.6 (0.9–2.0)	0.53 (0.40–0.58)	2.2 (1.4–3.8)	0.4 (0.4–0.5)	6.4 (5.0–8.0)	6265 (1306–12,152)
DT + 1	4.4 (3.9–4.7)	3.0 (2.5–3.2)	0.8 (0.6–1.1)	0.44 (0.30–0.60)	4.0 (2.2–5.3)	0.5 (0.5–0.6)	6.9 (5.4–9.5)	1099 (159–19,494)
DT + 2	3.3 (2.9–4.0)	1.9 (1.7–2.5)	0.7 (0.6–1.2)	0.32 (0.24–0.49)	2.6 (1.7–3.7)	0.5 (0.3–0.6)	6.2 (4.1–8.0)	100 (1–20,337)
DT + 3	3.4 (2.7–3.7)	1.5 (1.3–1.9)	1.0 (0.8–1.3)	0.36 (0.28–0.60)	1.6 (1.1–2.2)	0.5 (0.3–0.6)	3.6 (2.8–5.4)	1 (0–305)
Day 35	6.2 (5.2–7.2)	2.8 (2.3–3.3)	2.2 (1.9–3.0)	0.53 (0.44–0.71)	1.2 (0.9–1.3)	0.2 (0.2–0.2)	5.6 (4.1–6.4)	0 (0–0)
p value	< 0.0001	< 0.0001	< 0.0001	0.002	< 0.0001	< 0.0001	< 0.0001	< 0.0001

Data in this table are not normally distributed and shown as median (interquartile range). p values in this table were derived from Friedman tests

Linear model analysis showed that in the individuals who develop parasitaemia, the leukocyte count remained stable until DT − 1 and then showed a fall, which continued until it reached its nadir on DT + 2, with a median of 3.3 × 10^5^ leukocytes/L. On day DT + 2, a leukocytopenia was observed in 70% (14/20) of the subjects. The absolute neutrophil count started to decrease on DT − 1 to a median value of 1.5 × 10^5^ neutrophils/L on DT + 3. Neutropenia was found in 40% (8/20) of the subjects on this day. A steady fall in absolute lymphocyte count was observed from DT − 2 until DT + 2, when the median lymphocyte count was 0.7 × 10^5^ lymphocytes/L. The percentage of subjects with an absolute lymphocytopenia increased from 30% (6/20) on DT to 70% (14/20) 2 days later. The median absolute monocyte counts remained within the normal range, but showed a mild drop on DT + 2. On day 35 of the study protocol, all parameters had returned to baseline values. The evaluated parameters did not differ significantly between subgroups of subjects infected with NF166.C8 and NF135.C10.

**Fig. 2:**
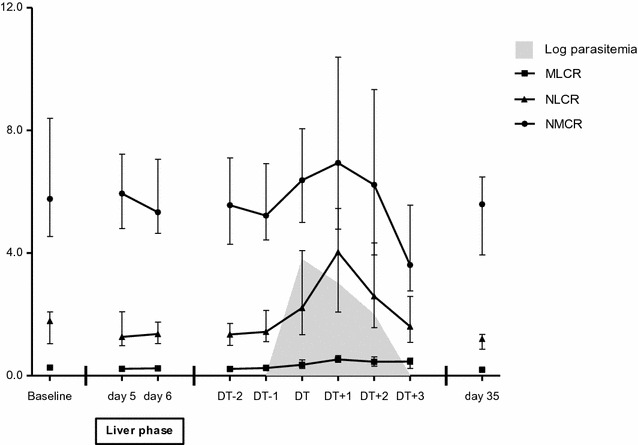
Changes in differential cell count ratios in the 20 subjects who developed malaria in CHMI-b. The data are shown as medians (dots) and interquartile ranges (whiskers). The data from DT − 2 until DT + 3 were synchronized on DT

The differential cell count ratios were also found to change significantly over time using the Friedman test (Table 1, Fig. 2). The linear model analysis showed a gradual rise of the NLCR, reaching a maximum of 4.0 on DT + 1. After DT + 1 a rapid decrease was observed. The MLCR showed a rise from DT − 2 to DT + 1 after which it decreased. The NMCR seemed to show a rise followed by a fall but these changes were not significant in the linear model analysis.

In the four subjects that remained qPCR negative throughout the study period, the differential leukocyte counts remained within normal values and no significant changes in cell counts or cell count ratios were observed. All four received a full course of atovaquone–proguanil at day 13 of the study protocol (Additional file 1: Table S1 and Additional file 3: Figure S2).

### Correlations

Significant correlations were found between parasite load and absolute lymphocyte count (p < 0.001, correlation coefficient − 0.46) and between parasite load and NLCR (p < 0.001, correlation coefficient 0.50) in CHMI-b. Correlations between parasite load and total leukocyte count, MLCR and NMCR were significant, but weak (p = 0.004, Spearman’s rho (r_s_) − 0.23, p < 0.001, r_s_ 0.36 and p = 0.003, r_s_ 0.23, respectively). There was no significant correlation between parasite load and absolute neutrophil count or absolute monocyte count.

## Discussion

The present CHMI study shows an increase in total leukocyte, lymphocyte and monocyte count during parasite liver stages of infected subjects. Upon blood stage infection, a decrease is observed in total leukocyte, neutrophil, lymphocyte and monocyte count; these changes clearly relate to the occurrence of blood parasitaemia and are not caused by treatment with atovaquone–proguanil, as shown by the control group of four subjects, who remained non-parasitaemic until day 13 after mosquito exposure and were then treated with a full course of atovaquone–proguanil. Although it cannot be excluded that one or more of these four non-parasitaemic subjects had a liver stage infection when treatment was initiated, we regard it more likely that no sporozoite transmission occurred. In this group, no changes in total or differential leukocyte counts were observed during the study period.

The increase of the total leukocyte count, lymphocytes and monocytes during liver stage development has not been previously reported. During the clinically silent liver phase of malaria, only a relatively low number of hepatocytes become infected and the host immune responses to this stage of the parasite are poorly understood [24]. Previous CHMI studies have studied inflammatory parameters and cellular and humoral immune responses, but only demonstrated changes in these markers during the blood phase of the infection [15–20, 25]. The finding that the total leukocyte count, lymphocyte count and monocyte count show an increase during the liver phase is remarkable and further studies are needed to confirm this finding and to elucidate its underlying pathophysiological mechanism.

The most pronounced change after the liver phase is a fall in lymphocyte count that starts on DT − 1 and reaches its nadir at 0.7 × 10^5^ lymphocytes/L on DT + 2, when 65% of subjects are lymphocytopenic. The neutrophil count only starts to show a significant fall after DT. These changes are reflected in the NLCR, which shows a steep rise to 4.0 on DT + 2. In comparison, in a previous study on patients with imported malaria the median NLCR on admission of all patients was 3.2, and 3.5 in patients with a severe *P. falciparum* infection [12].

Lymphocytopenia has been described in several large cohorts of patients with malaria [9, 26–28] and is highly prevalent in symptomatic travelers with imported malaria [12]. The correlations between parasite and lymphocyte count and between parasite load and NLCR were also demonstrated in a study by Berens-Riha et al. [11], in which patients were stratified according to their immune status. Both the MLCR and the NLCR were found to be lower in semi-immune patients as compared to non-immune patients. In the present study, all subjects were non-immune. White blood cell count changes have also been described in previous CHMI studies. In a study on clinical manifestations in CHMI with *P. falciparum,* Church et al. described a decrease in total white blood cell count and in neutrophil count, but not in lymphocyte count, with the nadir occurring 2 days after therapy initiation [15]. A decrease in lymphocyte count was observed in several studies involving sporozoite infection after mosquito bite exposure [16–19] and in a study using experimental inoculation with a low number of parasitized erythrocytes [20]. These observations are in line with the findings described here. As no measurements are available between DT + 3 and the end of the study protocol at day 35, we are not able to monitor when the changes observed, started to normalize after treatment. This is an important limitation of the study.

Several mechanisms have been proposed as an explanation for malaria induced lymphocytopenia. A temporary reallocation of lymphocytes has been suggested, which is supported by the observation that lymphocytes rapidly re-emerge in the peripheral circulation after treatment is initiated [29]. Others suggest that the soluble Fas ligand induces apoptosis of lymphocytes, which has been observed both in vitro and in healthy donors from endemic areas [30–34], and a previous CHMI study found an increased production of Granzyme B, which is also know to induce apoptosis, in exposed individuals, [35]. Both mechanisms though might be partly responsible for the observed fall in lymphocyte cell count.

## Conclusions

In a setting of controlled human malaria infection in healthy non-immune volunteers, an initial increase of peripheral total leukocyte count and differential lymphocytes and monocytes was observed during liver stage infection of 35 infected volunteers. This rise seems to indicate the presence of a malaria infection and is followed 2–3 days later by the appearance of parasites in the peripheral blood, which is accompanied by a marked decrease in total leukocyte count, lymphocyte count and the neutrophil count and a rise of the NLCR. Both the lymphocyte count and the NLCR correlated with blood parasitaemia, and all parameters had normalized 3–4 weeks after parasite clearance. The non-parasitaemic control group in this study excludes a treatment-related effect.

## Additional files

**Additional file 1: Table S1.** Differential leukocyte counts and their ratios in the 4 non-parasitemic subjects in CHMI-b.

**Additional file 2: Figure S1.** Changes in total and differential leukocyte counts in the 4 subjects who did not develop malaria in CHMI-b. The data are shown as medians (dots) and interquartile ranges (whiskers). None of the changes in this group were statistically significant.

**Additional file 3: Figure S2.** Changes in differential cell count ratios in the 4 subjects who did not develop malaria in CHMI-b. The data are shown as medians (dots) and interquartile ranges (whiskers). None of the changes in this group were statistically significant.

## Abbreviations

CHMI: controlled human malaria infection
NLCR: neutrophil to lymphocyte count ratio
MLCR: monocyte to lymphocyte count ratio
NMCR: neutrophil to monocyte count ratio
qPCR: quantitative polymerase chain reaction
