# Supplementary material for: Changes in total and differential leukocyte counts during the clinically silent liver phase in a controlled human malaria infection in malaria-naïve Dutch volunteers
Source: Malar J. 2017 Nov 10;16:457. doi: 10.1186/s12936-017-2108-1 (PMC5681833; doi:10.1186/s12936-017-2108-1)
Supplement: Supplementary file 1 — Additional file 1: Table S1. Differential leukocyte counts and their ratios in the 4 non-parasitemic subjects in CHMI-b. [file 12936_2017_2108_MOESM1_ESM.docx]

|  | Leukocytes  X10^9^/L | Neutrophils  X10^9^/L | Lymphocytes  X10^9^/L | Monocytes  X10^9^/L | NLCR | MLCR | NMCR |  |
| --- | --- | --- | --- | --- | --- | --- | --- | --- |
| Baseline | 6.3 (6.2-6.8) | 3.9 (3.7-4.2) | 1.8 (1.8-2.0) | 0.54 (0.45-0.62) | 2.1 (2.0-2.3) | 0.3 (0.2-0.3) | 8.1 (6.0-10.3) |  |
| Day 5 | 6.4 (6.1-6.8) | 3.6 (3.1-4.0) | 2.2 (2.1-2.3) | 0.57 (0.51-0.63) | 1.6 (1.4-2.0) | 0.3 (0.2-0.3) | 6.6 (5.5-7.5) |  |
| Day 6 | 6.6 (6.4-7.2) | 3.8 (3.6-4.0) | 2.1 (1.9-2.5) | 0.53 (0.51-0.62) | 1.7 (1.5-1.9) | 0.3 (0.3-0.3) | 6.9 (6.3-7.1) |  |
| Day 7 | 6.1 (5.9-7.1) | 3.5 (3.2-4.5) | 1.9 (1.8-2.1) | 0.57 (0.52-0.59) | 2.0 (1.7-2.4) | 0.3 (0.2-0.3) | 7.4 (6.4-8.9) |  |
| Day 8 | 6.6 (6.4-6.8) | 3.5 (3.4-3.8) | 2.1 (2.0-2.2) | 0.56 (0.53-0.60) | 1.6 (1.4-1.8) | 0.3 (0.2-0.3) | 6.6 (6.0-7.0) |  |
| Day 9 | 6.0 (5.1-7.2) | 3.5 (2.9-4.4) | 1.9 (1.7-2.1) | 0.50 (0.46-0.51) | 2.0 (1.8-2.2) | 0.2 (0.2-0.3) | 8.0 (6.8-9.1) |  |
| Day 10 | 6.4 (5.8-7.3) | 3.7 (3.3-4.2) | 2.2 (1.9-2.4) | 0.57 (0.49-0.66) | 1.7 (1.6-1.9) | 0.3 (0.3-0.3) | 6.5 (6.4-6.7) |  |
| Day 11 | 6.0 (5.8-6.2) | 3.3 (2.9-3.6) | 2.1 (2.0-2.2) | 0.47 (0.42-0.52) | 1.6 (1.3-1.9) | 0.2 (0.2-0.3) | 7.4 (6.6-7.7) |  |
| Day 12 | 5.6 (5.6-5.9) | 3.2 (3.0-3.6) | 1.8 (1.7-1.9) | 0.47 (0.42-0.51) | 1.9 (1.7-2.0) | 0.3 (0.2-0.3) | 7.2 (6.6-8.2) |  |
| Day 13^ | 5.8 (5.5-6.0) | 3.2 (3.0-3.3) | 1.9 (1.8-1.9) | 0.44 (0.39-0.50) | 1.6 (1.5-1.8) | 0.2 (0.2-0.2) | 6.7 (6.6-7.0) |  |
| Day 14 | 6.2 (5.5-6.4) | 3.7 (3.1-3.9) | 1.8 (1.7-1.8) | 0.39 (0.35-0.46) | 2.0 (1.7-2.2) | 0.2 (0.2-0.3) | 8.3 (6.3-10.5) |  |
| Day 15 | 5.7 (5.0-6.3) | 3.3 (3.0-3.5) | 1.6 (1.5-1.8) | 0.43 (0.40-0.45) | 2.0 (1.6-2.3) | 0.2 (0.2-0.3) | 7.9 (6.5-9.4) |  |
| Day 16 | 6.8 (6.3-6.9) | 3.8 (3.2-4.2) | 2.0 (1.9-2.1) | 0.53 (0.50-0.56) | 1.8 (1.4-2.2) | 0.3 (0.2-0.3) | 6.5 (6.0-7.6) |  |
| End | 7.2 (6.7-7.7) | 3.9 (3.6-4.4) | 2.2 (1.9-2.4) | 0.58 (0.43-0.77) | 2.1 (1.8-2.3) | 0.3 (0.3-0.3) | 7.0 (5.6-8.5) |  |
| p-value | 0.21 | 0.66 | 0.10 | 0.08 | 0.28 | 0.74 | 0.65 |  |
| Data are shown as median (interquartile range). P-values in this table were derived from Friedman tests. ^Non-parasitemic subjects received a full 3-day course of atovaquone / proguanil, starting on day 13. | | | | | | | | |

**Additional table S1:** Differential leukocyte counts and their ratios in the 4 non-parasitemic subjects in CHMI-b.
