# Supplementary material for: Changes in total and differential leukocyte counts during the clinically silent liver phase in a controlled human malaria infection in malaria-naïve Dutch volunteers
Source: Malar J. 2017 Nov 10;16:457. doi: 10.1186/s12936-017-2108-1 (PMC5681833; doi:10.1186/s12936-017-2108-1)

**Additional figure S1: Changes in total and differential leukocyte counts in the 4 subjects who did not develop malaria in CHMI-b.** The data are show as medians (dots) and interquartile ranges (whiskers). None of the changes in this group were statistically significant.


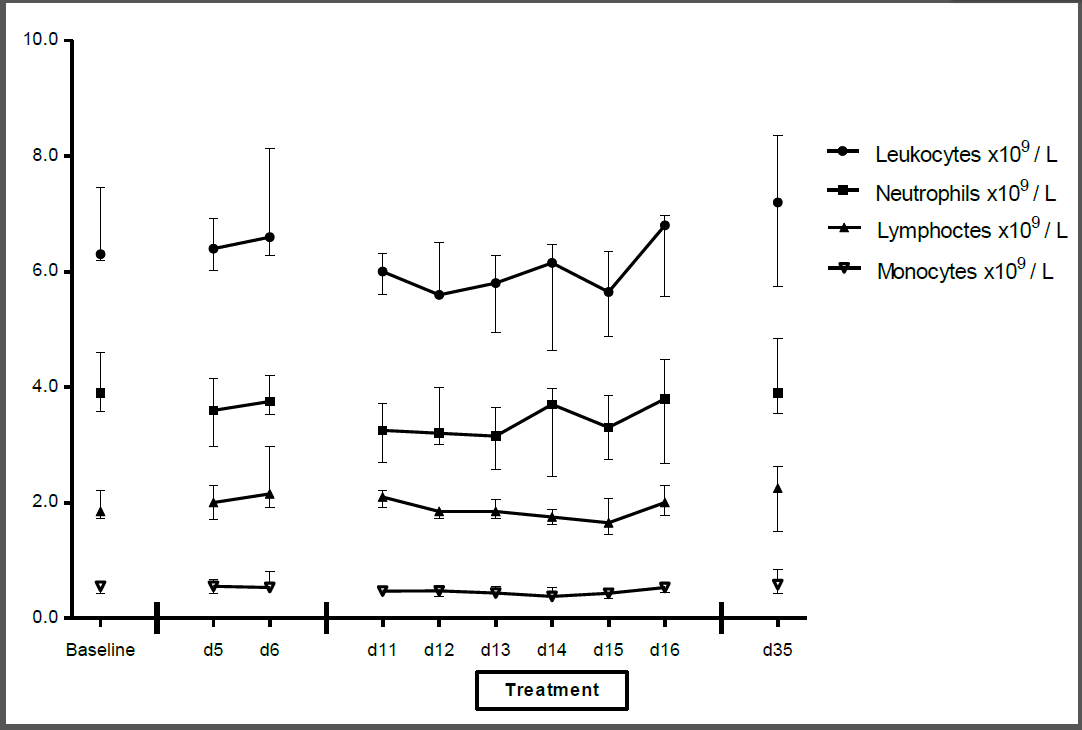

Supplement: Supplementary file 2 — Additional file 2: Figure S1. Changes in total and differential leukocyte counts in the 4 subjects who did not develop malaria in CHMI-b. The data are shown as medians (dots) and interquartile ranges (whiskers). None of the changes in this group were statistically significant. [file 12936_2017_2108_MOESM2_ESM.docx]
